# Supplementary material for: The Wnt/TCF7L1 transcriptional repressor axis drives primitive endoderm formation by antagonizing naive and formative pluripotency
Source: Nat Commun. 2023 Mar 3;14:1210. doi: 10.1038/s41467-023-36914-1 (PMC9984534; doi:10.1038/s41467-023-36914-1)
Supplement: Supplementary file 1 — Supplementary Information [file 41467_2023_36914_MOESM1_ESM.pdf]

Supplementary Information

The Wnt/TCF7L1 transcriptional repressor axis drives primitive endoderm formation by antagonizing naive and formative pluripotency

Paraskevi Athanasouli [1,10], Martina Balli [1,10], Anchel De Jaime-Soguero [1,*], Annekatrien Boel [2], Sofia Papanikolaou [3,4], Bernard K. van der Veer [1], Adrian Janiszewski [1], Tijs Vanhessche [1], Annick Francis [5], Youssef El Laithy [1], Antonio Lo Nigro [1], Francesco Aulicino [6], Kian Peng Koh [1], Vincent Pasque [1,7], Maria Pia Cosma [6,8,9], Catherine Verfaillie [1], An Zwijsen [5], Björn Heindryckx [2], Christoforos Nikolaou [4], and Frederic Lluis[1,*]

[1] KU Leuven, Department of Development and Regeneration, Stem Cell Institute, B-3000, Leuven, Belgium

[2] Ghent-Fertility And Stem cell Team (G-FaST), Department for Reproductive Medicine, Department for Human Structure and Repair, Ghent University Hospital, 9000, Ghent, Belgium

[3] Department of Rheumatology, Clinical Immunology, Medical School, University of Crete, 70013, Heraklion, Greece

[4] Computational Genomics Group, Institute of Bioinnovation, Biomedical Sciences Research Center "Alexander Fleming", 16672, Athens, Greece

[5] Department of Cardiovascular Sciences, KU Leuven, 3000, Leuven, Belgium

[6] Centre for Genomic Regulation (CRG), Dr Aiguader 88, 08003, Barcelona, Spain

[7] KU Leuven Institute for Single-Cell Omics (LISCO), 3000, Leuven, Belgium

[8] ICREA, Pg. Lluis Companys 23, Barcelona 08010, Spain

[9] Universitat Pompeu Fabra (UPF), Barcelona, Spain

[10] These authors contributed equally

*email: anchel.dejaime@cos.uni-heidelberg.de
Frederic.lluisvinas@kuleuven.be

Supplementary Figure 1

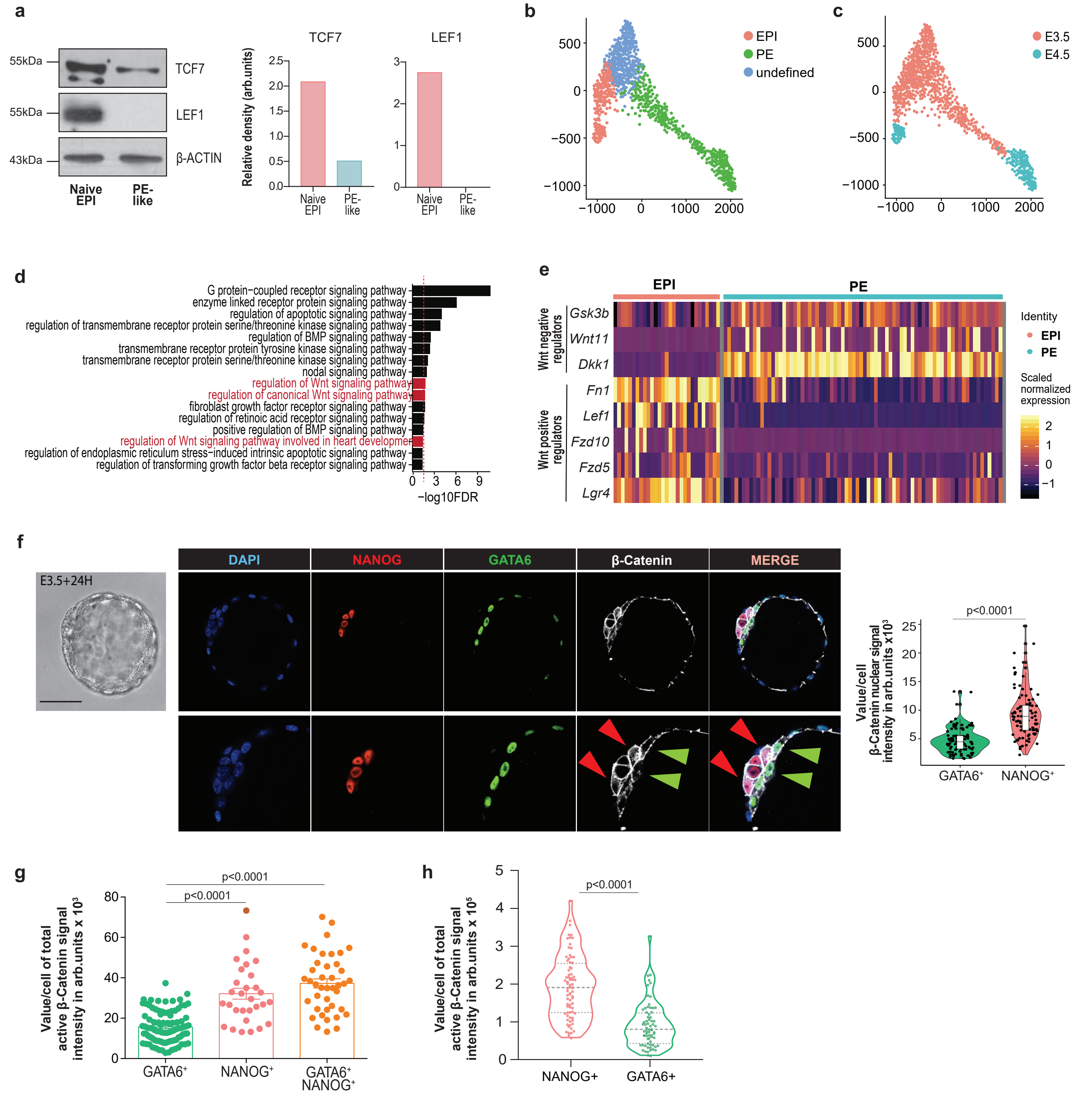

Supplementary Fig. 1: PE cells are characterized by a diminished Wnt/β-Catenin signaling activity compared to the EPI lineage.
(a) TCF7 and LEF1 protein levels in naive EPI and PE-like cells from 1 experiment. **(b)** Force-directed layout displaying relationships between undefined ICM, EPI and PE cells. Cells are colored by lineage [50]. **(c)** Force-directed layout of E3.5 and E4.5 cells. Cells are colored by embryo developmental timepoint [50]. **(d)** GO enrichment analysis of DEGs between EPI and PE lineages in E4.5 preimplantation embryos [49]. Red dashed line=FDR 0.05. **(e)** Differential Wnt signaling transcriptome profile between EPI and PE lineages in E4.5 preimplantation embryos [49]. **(f)** (Left) Representative immunofluorescence (IF) image of active β-Catenin protein signal in E3.5+24H *ex vivo* cultured embryos. EPI-NANOG$^+$ cells (red arrow) and PE-GATA6$^+$ cells (green arrow). Scale bar=50μM. Zoomed region of interest is reported below the images. (Right) Nuclear active β-Catenin signal intensity in EPI-NANOG$^+$ and PE-GATA6$^+$. NANOG$^+$ n=91, GATA6$^+$ n=100; 2 independent experiments; two-tailed unpaired *t* test. Horizontal line denotes the median value, box indicates the interquartile range (IQR) and whiskers denote the 1.5 × IQR. **(g)** Total active β-Catenin signal intensity in EPI-NANOG$^+$, PE-GATA6$^+$ and double-positive NANOG$^+$/GATA6$^+$ cells in E3.5 embryos. Mean ± SEM; NANOG$^+$ n=30, GATA6$^+$ n=93 and undefined NANOG$^+$/GATA6$^+$ cells n=41; 2 independent experiments; one-way ANOVA test. **(h)** Total active β-Catenin signal intensity in EPI-NANOG$^+$ and PE-GATA6$^+$ cells. Integrated intensity in arbitrary units (arb. units). NANOG$^+$ n=76; GATA6$^+$ n=69; 2 independent experiments; two-tailed unpaired *t* test. Source data for all experiments are provided as a Source data file.

Supplementary Figure 2

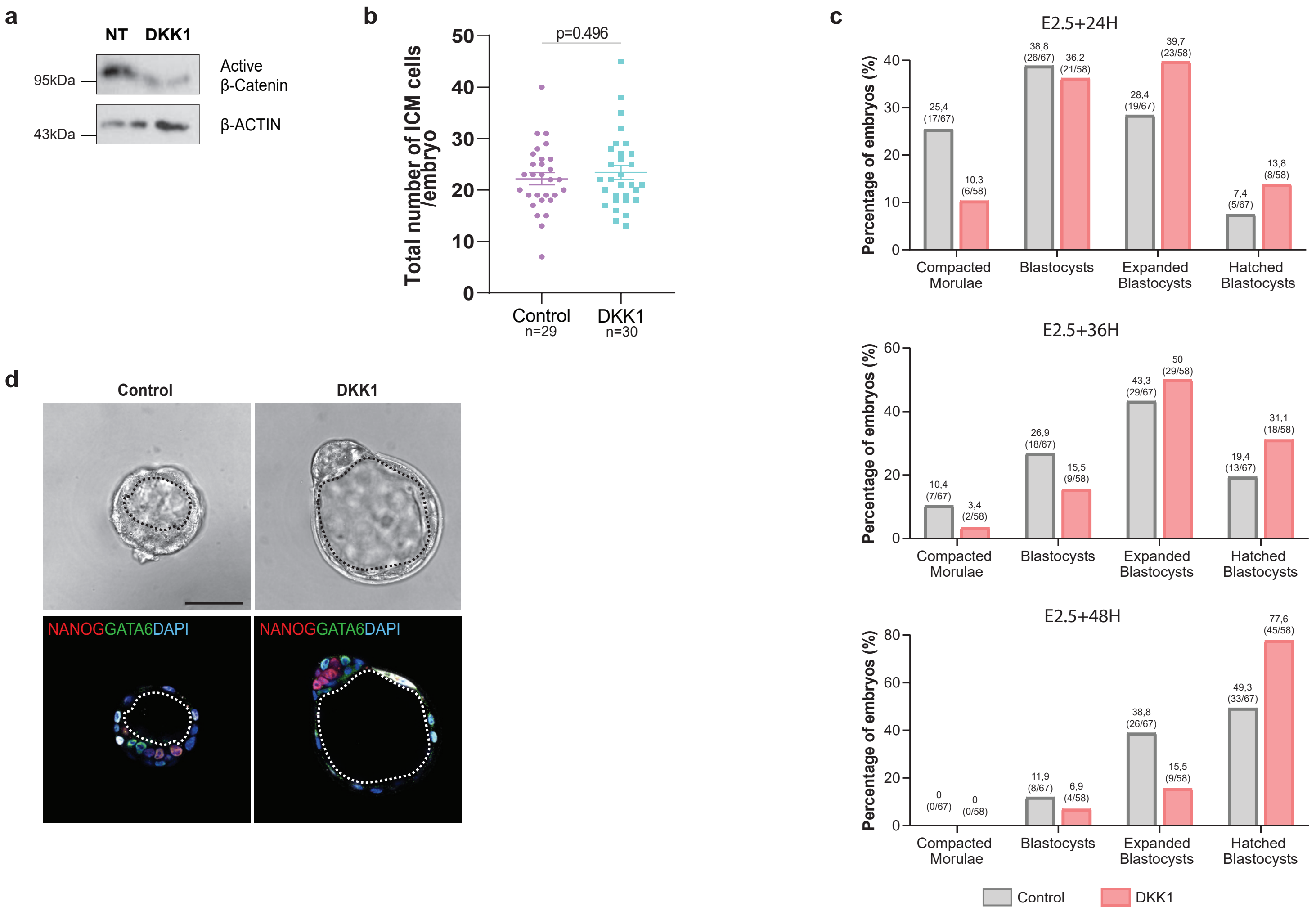

Supplementary Fig. 2: Wnt signaling inhibition as major contributor of PE lineage development.

(a) Representative images of active β-Catenin levels in mESCs upon DKK1 treatment for 3 passages from 3 independent experiments. **(b)** Total number of ICM cells per embryo. Mean ± SEM; Control: n=29, DKK1: n=30; 3 independent experiments; two-tailed unpaired *t* test. **(c)** Effect of DKK1 on mouse embryo developmental kinetics. Bars represent percentage of each developmental stage in each treatment group. Combined data of 2 *in vitro* culture sessions; see Source data file. **(d)** Representative IF image of embryo morphology upon DKK1 treatment from 3 independent experiments. Scale bar=50μM. Source data are provided in the Source data file.

Supplementary Figure 3

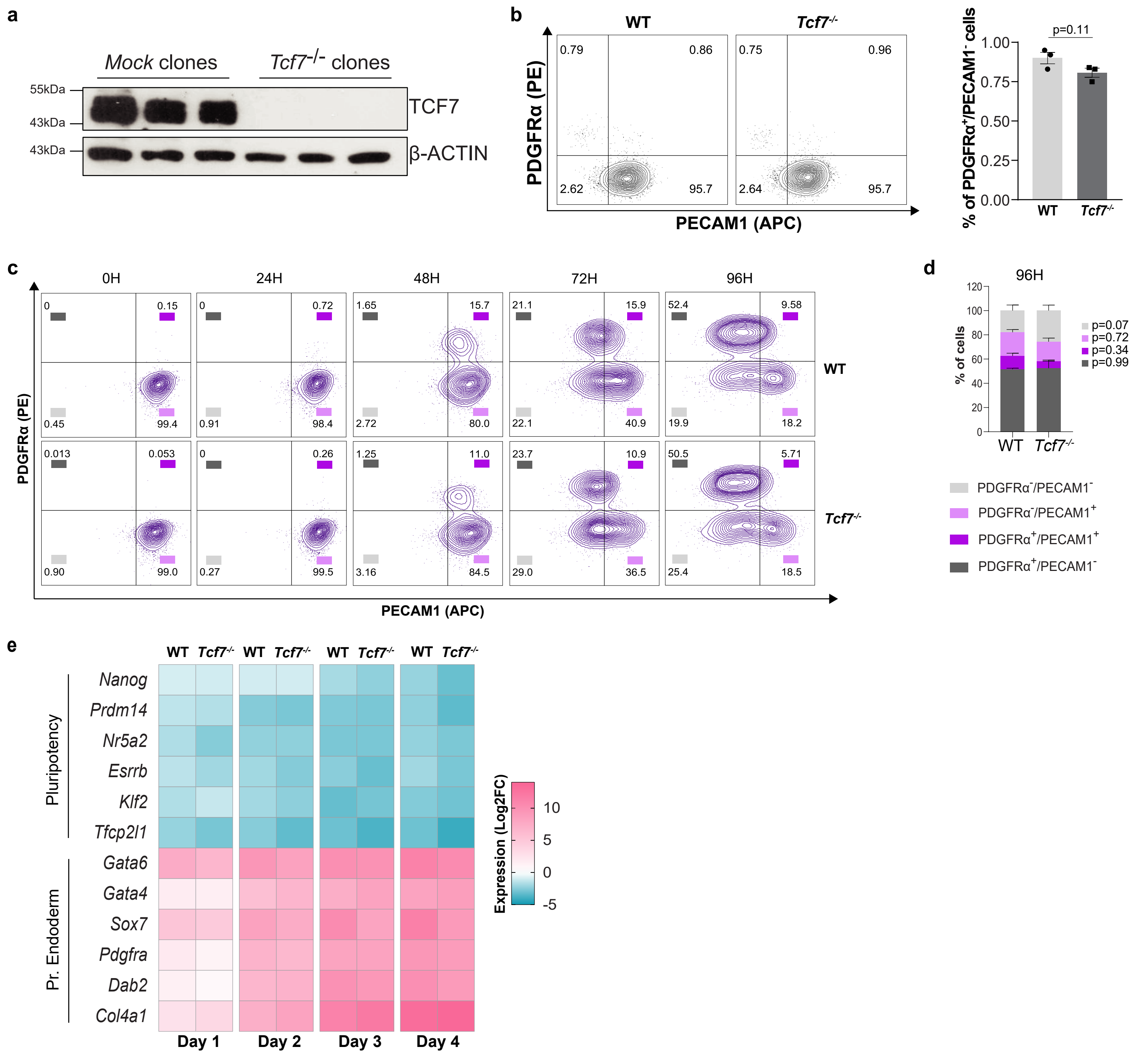

Supplementary Fig. 3: Loss of *Tcf7* does not affect PE specification.
(a) TCF7 protein levels in three independent mock and *Tcf7*$^{-/-}$ clones from 1 experiment. **(b)** (Left) Representative flow cytometry images of mESC populations co-stained for PECAM1 and PDGFRα in WT and *Tcf7*$^{-/-}$ cells. (Right) Flow cytometry analysis of PDGFRα^{+}/PECAM1^{-} cells in WT and *Tcf7*$^{-/-}$ cells. Mean ± SD; n=3 independent experiments. two-tailed unpaired *t* test. **(c)** Representative flow cytometric images of mESC populations co-stained for PECAM1 and PDGFRα markers in WT and *Tcf7*$^{-/-}$ cells upon 0, 24, 48, 72 and 96H of 0.25μM RA treatment. n=3. **(d)** Flow cytometry analysis of PDGFRα and PECAM1 populations at 96H of RA treatment. Mean ± SD; n=3 independent experiments; two-way ANOVA test. **(e)** Gene expression analysis of pluripotency and PE gene markers in WT and *Tcf7*$^{-/-}$ upon RA differentiation. Gene expression values are reported as Log_2 of fold change expression. Source data for all experiments are provided as a Source data file.

Supplementary Figure 4

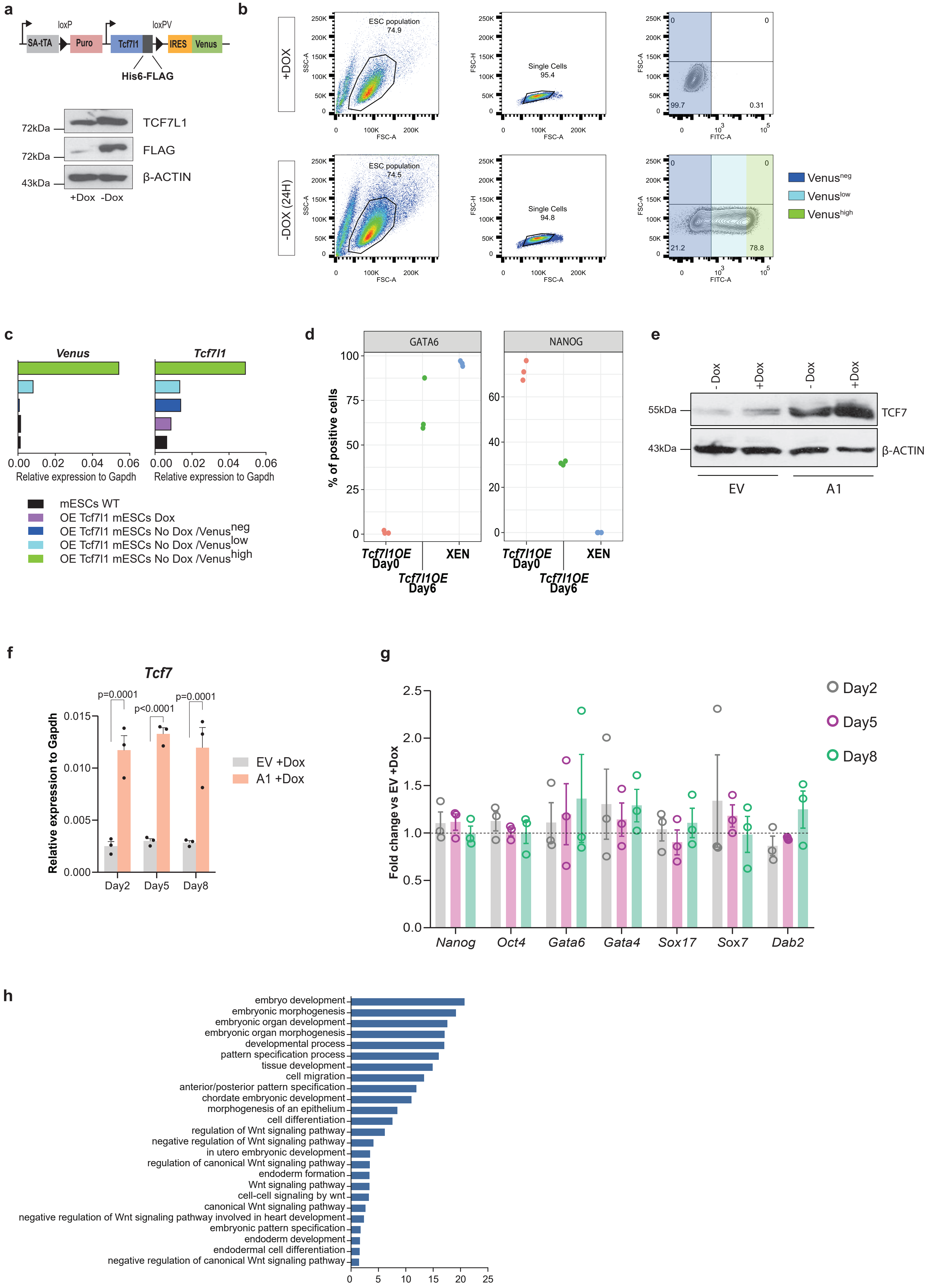

Supplementary Fig. 4: *Tcf7l1* overexpression promotes PE specification while TCF7 has negligible role in PE induction.

(a) (Upper panel) Schematic representation of *Tcf7l1* transgene as designed by Nishiyama et al. 2009. (Lower panel) TCF7L1 and FLAG protein levels in uninduced (+Dox) and 24h induced cells (-Dox). Representative of 3 independent experiments. **(b)** Venus/FITC expression in uninduced (+Dox) and 24H induced cells (-Dox). Exemplary sorting gating strategy for experiments presented in Fig. 4a-e, Supplementary Fig.4c,d and RNA sequencing samples. Colored areas separate negative, low and high *Venus*-expressing populations. $Venus^{high}$ population was sorted (last panel) **(c)** qRT-PCR of *Venus* and *Tcf7l1* in sorted cell populations after 24h of transgene induction. Populations were sorted based on *Venus* expression into $Venus^{neg}$, $Venus^{low}$ and $Venus^{high}$. WT mESCs and uninduced (+Dox) cells were used as control. Gene expression was normalized to *Gapdh*. **(d)** Percentage of GATA6 and NANOG positive nuclei normalized on the total number of cells (DAPI signal); see Source data file. Each dot represents a field of view. **(e)** TCF7 levels upon transgene induction with Dox treatment. EV: empty vector, A1: cell clone with stably incorporated transgene cassette. Representative of 3 independent experiments.**(f)** qRT-PCR of *Tcf7* upon transgene induction for 2, 5 and 8 days. EV treated with Dox was used as control. Mean ± SD; n=3 independent experiments; see Source data file. Two-way ANOVA test. **(g)** qRT-PCR of pluripotency and PE genes normalized to *Gapdh* upon *Tcf7* overexpression for 2, 5 and 8 days. Values are reported as fold change relative to EV treated with Dox. Mean ± SEM; n=3 independent experiments; see Source data file; multiple unpaired *t* tests with Holm-Sidak method; no statistically significant differences detected. **(h)** GO enrichment analysis of upregulated genes after 8 days of *Tcf7l1 OE*.

Supplementary Figure 5

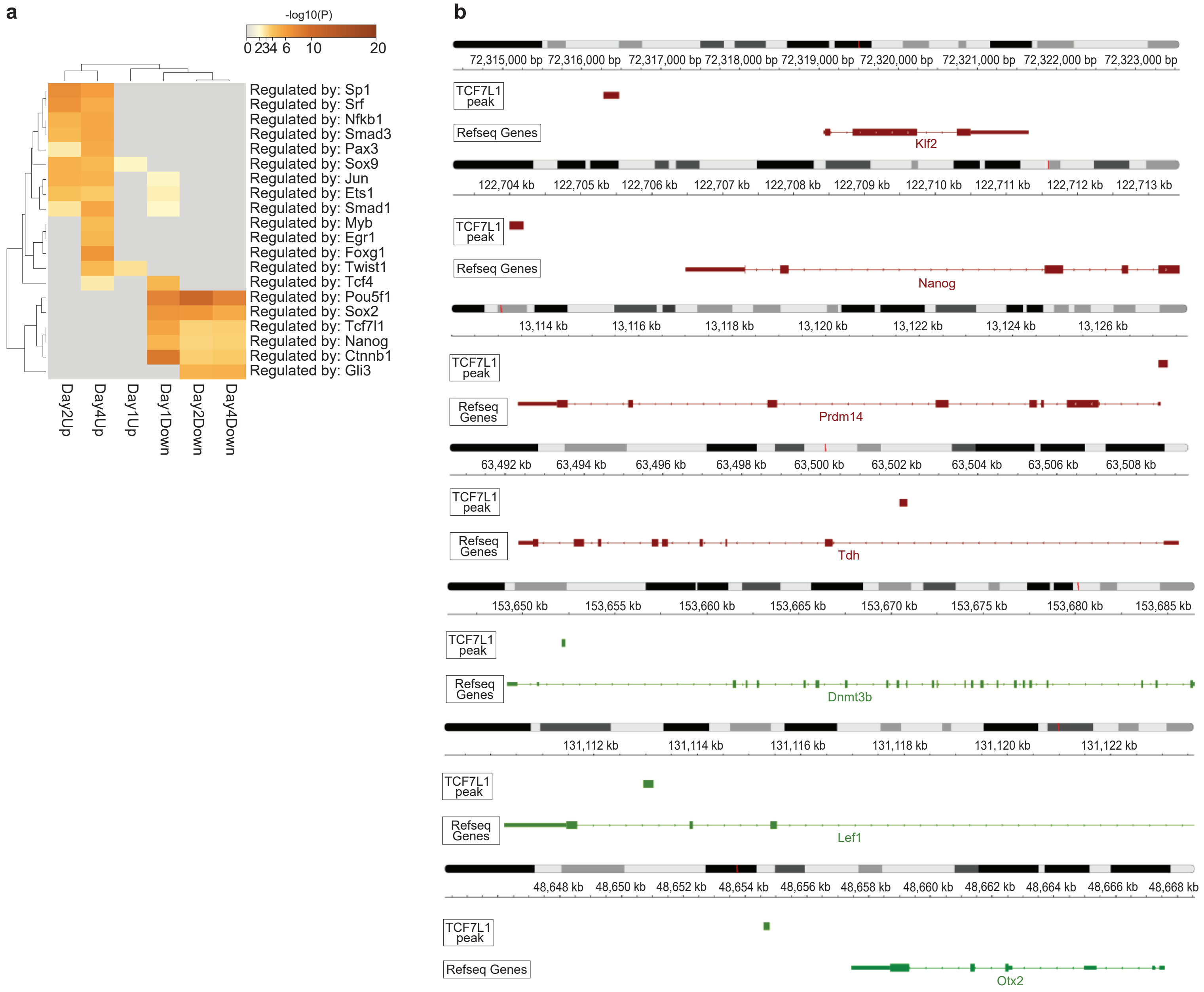

Supplementary Fig. 5 *Tcf7l1* overexpression promotes conversion to PE fate through a downregulation of naive and formative pluripotency.
(a) Transcriptional regulator target gene enrichments for genes up- or down-regulated at D1, D2 and D4 of *Tcf7l1* induction. Genes were selected at $|logFC| \geq 2$ and adjusted p-value≤ 0.05.
(b) Genomic regions around genes associated with naive, general and formative pluripotency genes with TCF7L1 binding peaks around the TSS region.

Supplementary Figure 6

a

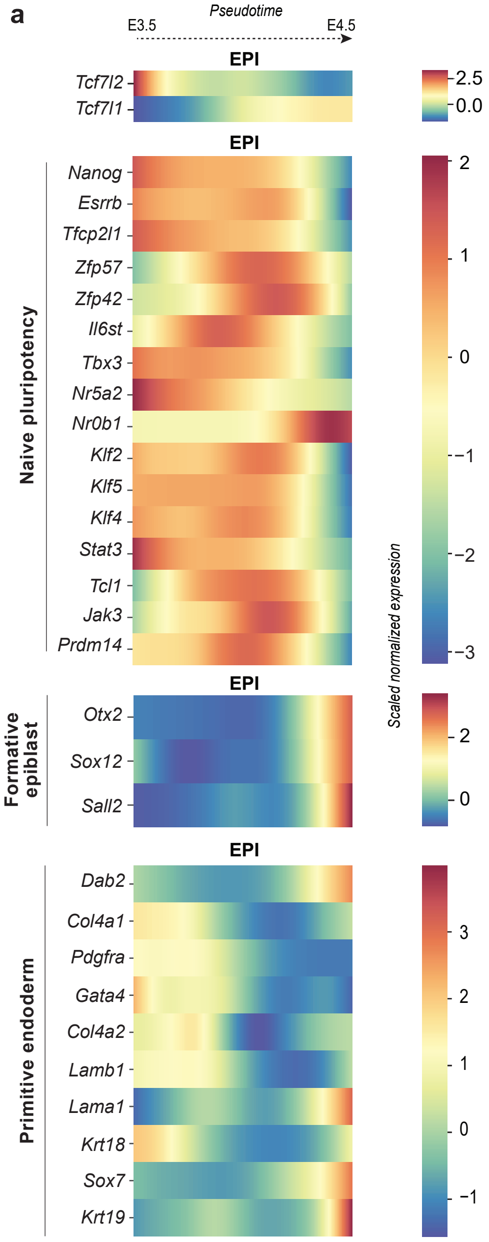

b

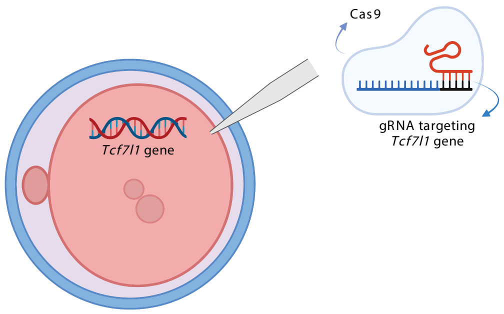

c

CRISPR/Ca9 Tcf7l1-/- embryos	MiSeq editing frequency	MiSeq predominant mutation			
		Chromosome	Positions (mm10)	Type	Length
**Embryo1 Tcf7l1-/-**	100%	chr6	72788276_72788332	DEL	57
		chr6	72788203_72788287	DEL	85
**Embryo2 Tcf7l1-/-**	100%	chr6	72788277_72788290	DEL	14
		chr6	72788279_72788278	INS	1
		chr6	72788279_72788278	INS	2
		chr6	72788273_72788381	DEL	109
**Embryo3 Tcf7l1-/-**	100%	chr6	72788262_72788299	DEL	38
**Embryo4 Tcf7l1-/-**	100%	chr6	72788278_72788281	DEL	4
		chr6	72788279_72788284	DEL	6
**Embryo5 Tcf7l1-/-**	100%	chr6	72788278_72788458	DEL	181
**Embryo6 Tcf7l1-/-**	100%	chr6	72788231_72788298	DEL	68
		chr6	72788278_72788281	DEL	4
**Embryo7 Tcf7l1-/-**	100%	chr6	72788277_72788290	DEL	14
		chr6	72788156_72788273	DEL	118
		chr6	72788270_72788458	DEL	189
**Embryo8 Tcf7l1-/-**	100%	chr6	72788249_72788284	DEL	36
		chr6	72788274_72788320	DEL	47
**Embryo9 Tcf7l1-/-**	100%	chr6	72788269_72788278	DEL	10
		chr6	72788280_72788282	DEL	3
**Embryo10 Tcf7l1-/-**	100%	chr6	72788278_72788281	DEL	4
		chr6	72788280_72788356	DEL	77
**Embryo11 Tcf7l1-/-**	100%	chr6	72788278_72788281	DEL	4
**Embryo12 Tcf7l1-/-**	100%	chr6	72788269_72788282	DEL	14
**Embryo13 Tcf7l1-/-**	100%	chr6	72788278_72788281	DEL	4
		chr6	72788279_72788284	DEL	6
		chr6	72788279_72788369	DEL	91
**Embryo14 Tcf7l1-/-**	100%	chr6	72788278_72788303	DEL	26
		chr6	72788279_72788326	DEL	48
**Embryo15 Tcf7l1-/-**	100%	chr6	72788262_72788290	DEL	29
		chr6	72788277_72788290	DEL	14

Supplementary Fig. 6 TCF7L1 as main regulator of *in vivo* PE cell fate specification. **(a)** *Tcf7l1* and *Tcf7l2* gene expression profile in EPI lineage from E3.5 to E4.5 compared to gene expression levels of different lineage markers. **(b)** Schematic representation of CRISPR/Cas9 *in vivo* experiment. Created with BioRender.com. **(c)** MiSeq results of CRISPR/Cas9 *Tcf7l1*$^{-/-}$ embryos.

Supplementary Figure 7

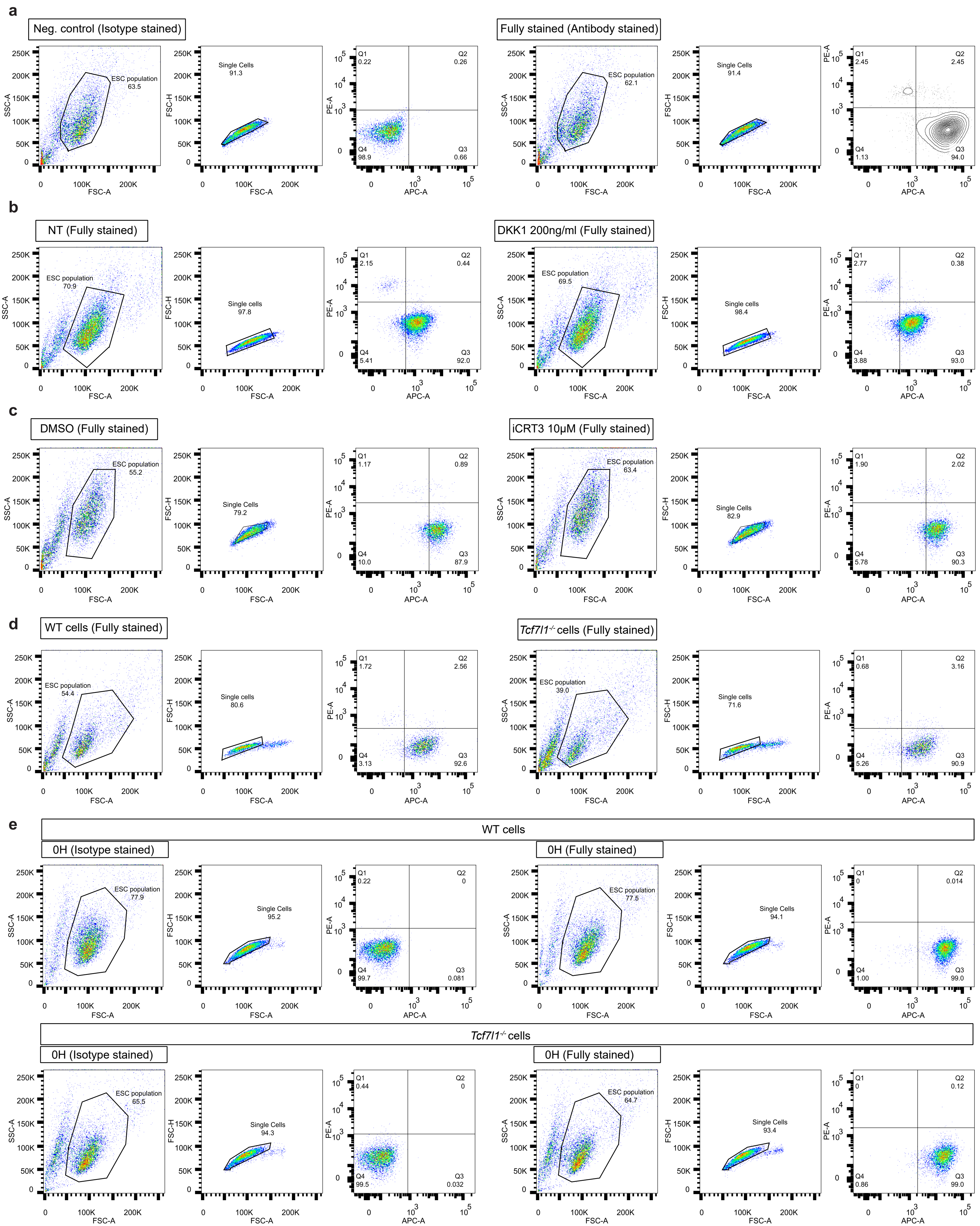

Supplementary Fig. 7: Gating strategies employed for FACS plots presented in the manuscript figures.

(a) Exemplary gating strategy to identify naive EPI (PECAM1$^+$/PDGFRα$^-$) and PE-like populations (PECAM1$^-$/PDGFRα$^+$) used in Fig.1b-e and Supplementary Fig. 1a. **(b)** Exemplary gating strategy to identify the PE-like population (PECAM1$^-$/PDGFRα$^+$) in NT and DKK1 treated cells presented in Fig. 2a. **(c)** Exemplary gating strategy to identify the PE-like population (PECAM1$^-$/PDGFRα$^+$) in DMSO and iCRT3 treated cells presented in Fig. 3a. **(d)** Exemplary gating strategy to identify the PE-like population (PECAM1$^-$/PDGFRα$^+$) in WT and *Tcf7l1*$^{-/-}$ or *Tcf7*$^{-/-}$ cells presented in Fig. 3c,d and Supplementary Fig. 3b. **(e)** Exemplary gating strategy to identify naive EPI (PECAM1$^+$/PDGFRα$^-$), PE-like (PECAM1$^-$/PDGFRα$^+$) and the intermediate populations in WT and *Tcf7l1*$^{-/-}$ or *Tcf7*$^{-/-}$ cells during RA timecourse presented in Fig. 3e,f and Supplementary Fig. 3c,d.

Supplementary Figure 8

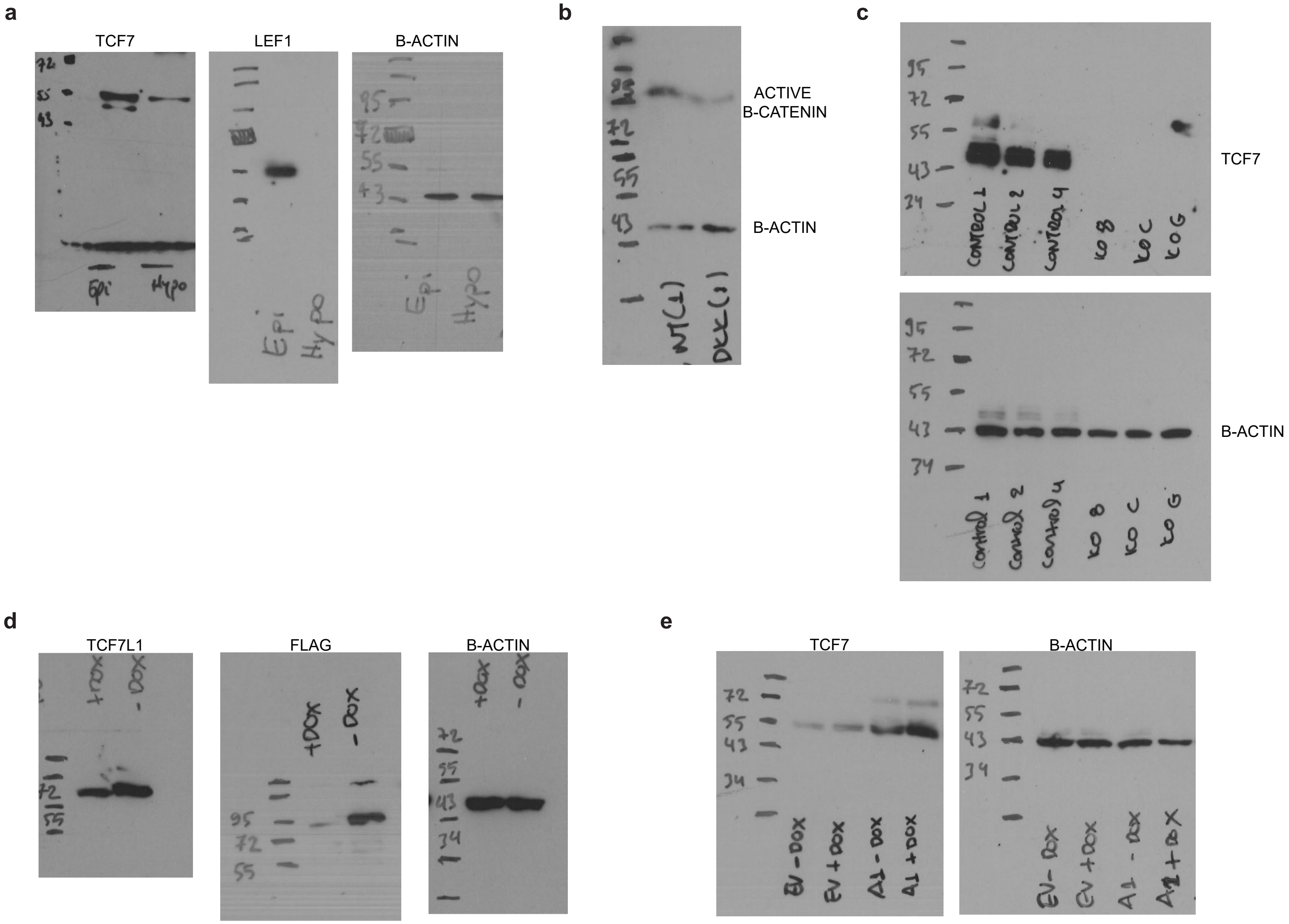

Supplementary Fig. 8: Uncropped and unprocessed scans of blots presented in Supplementary Figures.

Supplementary Table 1: Antibodies

NAME	SOURCE	IDENTIFIER
**Antibodies**		
Rat monoclonal anti-Nanog	eBioscience™	Cat#14-5761-80; RRID:AB_763613
Goat polyclonal anti-Gata6	R&D systems	Cat#AF1700, RRID:AB_2108901
Mouse monoclonal anti-Gata4	BD Biosciences	Cat#560327, RRID:AB_1645188
Mouse monoclonal anti-Cdx2	Biogenex	Cat#AM392GP
Rabbit monoclonal anti-(active)-β-catenin	Cell Signaling Technology	Cat#8814, RRID:AB_11127203
Goat polyclonal anti-Tcf3 (M-20) (Tcf7l1)	Santa Cruz Biotechnology	Cat#sc-8635, RRID:AB_2199133
Goat polyclonal anti-Nestin	Santa Cruz Biotechnology	Cat#sc-21248, RRID: AB_2148925
Mouse monoclonal anti-β-catenin	BD Biosciences	Cat#610154, RRID:AB_397555
Rabbit monoclonal anti-Tcf1 (C63D9)	Cell Signaling Technology	Cat#2203, RRID:AB_2199302
Rabbit monoclonal anti-Lef1 (C12A5)	Cell Signaling Technology	Cat#2230, RRID:AB_823558
Mouse anti-β-Actin (C4)	Santa Cruz Biotechnology	Cat#sc-47778, RRID: AB_626632
PE anti-mouse CD140a (PDGFRA) (APA5)	Thermo Fisher Scientific	Cat#12-1401-81, RRID:AB_657615
APC anti-mouse CD31 (PECAM-1) (390)	Thermo Fisher Scientific	Cat#17-0311-82, RRID:AB_657735
PE Rat IgG2α, κ Isotype Clone R35-95 (RUO)	BD Biosciences	Cat#553930, RRID:AB_479719
APC Rat IgG2α, κ Isotype (eBR2a)	Thermo Fisher Scientific	Cat#17-4321-81, RRID:AB_470181

Supplementary Table 2: Reagents and resources

NAME	SOURCE	IDENTIFIER
**Chemicals, Peptides, and Recombinant Proteins**		
XAV939	Sigma Aldrich	Cat#X3004
PD0325901	Sigma Aldrich	Cat#PZ0162
CHIR99021	Sigma Aldrich	Cat#SML1046
Retinoic acid	Sigma Aldrich	Cat#R2625
iCRT3	Sigma Aldrich	Cat#SML0211
Doxycycline (Dox)	Sigma Aldrich	Cat#D9891
Phosphatase inhibitor Cocktail 2	Sigma Aldrich	Cat# P5726
Phosphatase inhibitor Cocktail 3	Sigma Aldrich	Cat# P0044
Protease inhibitor cocktail	Sigma Aldrich	Cat# P8340
Recombinant Murine LIF	Peprotech	Cat#250-02
Recombinant Human/Murine/Rat Activin A	Peprotech	Cat#120-14E
Recombinant Human FGF-basic	Peprotech	Cat#100-18B
Recombinant Human DKK-1	Peprotech	Cat#120-30
Chorionic gonadotropin human	Sigma Aldrich	Cat#CG10-1VL
Cook Blasto	Cook Ireland Ltd, Ireland	Cat# G46296
N2 Supplement	Thermo Fisher Scientific	Cat#17502048
B27 Supplement	Thermo Fisher Scientific	Cat#12587010
Neurobasal	Gibco	Cat#21103-049
DMEM/F12	Gibco	Cat#11320-074
DMEM	Gibco	Cat#41965–039
Knockout DMEM	Gibco	Cat#10829-018
Knockout serum replacement	Gibco	Cat#10828-028
Normal Donkey serum	Jackson ImmunoResearch	Cat#017-000-121, RRID:AB_2337258
Human Plasma Fibronectin	Millipore	Cat#FC010
EmbryoMax 0.1% Gelatin Solution	Sigma Aldrich	Cat#ES-006-B
Accutase solution	Sigma Aldrich	Cat#A6964
Trypsin 0.05%	Gibco	Cat#25300-054
Cell Dissociation Solution	Sigma Aldrich	Cat#C5914
EmbryoMax® M2 Medium	Sigma Aldrich	Cat#MR-015-D
EmbryoMax® KSOM Mouse Embryo Media	Sigma Aldrich	Cat#MR-121-D
RIPA buffer	Sigma Aldrich	Cat#R0278
**Critical Commercial Assays**		
GenElute™ Mammalian Total RNA Miniprep Kit	Sigma Aldrich	Cat#RTN350
On-Column DNase I Digestion Set	Sigma Aldrich	Cat#DNASE70
iScript™ cDNA Synthesis Kit	Bio-Rad	Cat#1708890
Arcturus® PicoPure® DNA Extraction Kit	Thermo Fisher Scientific	Cat#KIT0103
KAPA Stranded RNA-Seq Library Preparation Kit Illumina® Platforms	Roche	Cat#7962207001
Qubit dsDNA HS Assay Kit	Thermo Fisher Scientific	Cat#Q32854
**Experimental Models: Cell lines**		
WT ESC	Merrill et al. 2004	N/A
Tcf7l1 KO ESC	Merrill et al. 2004	N/A
Tcf7 KO ESC	De Jaime-Soguero et al. 2017	N/A
Tcf7l1 OE ESC	Nishiyama et al. 2009	N/A

**Experimental Models: Organisms/Strains**		
Mouse/CD-1	Envigo	030
Mouse/B6D2F1	Charles River	099
**Oligonucleotides**		
Primers for qRT-PCT	See Supplementary Data 11	Integrated DNA Technologies
Tcf7l1-targeting crRNA	See Supplementary Data 11	Integrated DNA Technologies
Tcf7l1 MiSeq primers	See Supplementary Data 11	Integrated DNA Technologies
**Software and Algorithms**		
PySCENIC v0.9.15	Sande et al. 2020	https://github.com/aertslab/pySCENIC
Seurat v3.0.0	Stuart et al. 2019	https://github.com/satijalab/seurat
Harmony v0.1.4	Nowotschin et al. 2019	https://github.com/dpeerlab/Harmony
Palantir v1.0.0	Setty et al. 2019	https://github.com/dpeerlab/Palantir
Cutadapt	Martin 2011	http://code.google.com/p/cutadapt/
HISAT2 v2.1.0	Kim et al. 2019	https://github.com/DaehwanKimLab/hisat2
FeatureCounts v2.0.1	Liao et al. 2014	http://subread.sourceforge.net
DESeq2 v1.28.1	Love et al. 2014	http://www.bioconductor.org/packages/release/bioc/html/DESeq2.html
ClueGO Cytoscape plug-in	Bindea et al. 2009	http://www.ici.upmc.fr/cluego/cluegoDownload.shtml
Metascape	Zhou et al. 2019	http://metascape.org.
GEM	N/A	http://groups.csail.mit.edu/cgs/gem/
g: Profiler	Raudvere et al. 2019	https://biit.cs.ut.ee/gprofiler
R v3.6.1	N/A	https://www.r-project.org/
IGV Genome Browser	Robinson et al. 2011	https://software.broadinstitute.org/software/igv/
QuantStudio™ Real-Time PCR Software v1.3	N/A	https://www.thermofisher.com/be/en/home/global/forms/life-science/quantstudio-6-7-flex-software.html
Harmony High-Content Imaging and Analysis Software v4.8	PerkinElmer	N/A
Leica Application Suite v3.7.4	Leica Microsystems	N/A
AxioVision SE64 Rel. 4.9.1	Zeiss	N/A
BATCH-GE	Boel et al. 2016	https://github.com/WouterSteyaert/BATCH-GE
FlowJo v10.7.1	BD Biosciences	N/A
ImageJ v1.8.0_172	N/A	https://imagej.nih.gov/
GraphPad Prism v6.0	N/A	https://www.graphpad.com/scientific-software/prism/
**Deposited data**		
RNA-seq	This paper	GEO: GSE171204
Chip-seq	De Jaime-soguero et al. 2017	E-MTAB-4358
Single-cell RNA-seq	Posfai et al. 2021	GSE145609
Single-cell RNA-seq	Mohammed at al. 2017	GSE100597
Single-cell RNA-seq	Nowotschin et al. 2019	GSE123046
