# Supplementary material for: The Wnt/TCF7L1 transcriptional repressor axis drives primitive endoderm formation by antagonizing naive and formative pluripotency
Source: Nat Commun. 2023 Mar 3;14:1210. doi: 10.1038/s41467-023-36914-1 (PMC9984534; doi:10.1038/s41467-023-36914-1)
Supplement: Supplementary file 3 — Description of Additional Supplementary Files [file 41467_2023_36914_MOESM3_ESM.pdf]

Description of Additional Supplementary Files

File name: Supplementary Data 1
Description: Gene ontology enrichment analysis from DEGs identified between naive EPI and PE-like populations identified by RNA-seq in Lo Nigro et al., 2017 (sheet: Gene ontology enrichment) relating to Figure 1c. The second sheet contains the GO terms shown in Figure 1c.

File name: Supplementary Data 2
Description: Differential gene expression analysis between EPI and PE populations identified by scRNA-seq of E4.5 embryos in Nowotschin et al., 2019 (sheet: DEGs E4.5 - DEG_EPIvsPE). Second sheet "GO from DEGs FDR<0,05" contains gene ontology enrichment analysis of DEGs identified in first sheet. Third sheet "GO pathways from DEGs FDR<0,05" contains gene ontology enrichment analysis of DEGs identified in first sheet narrowed down to signaling pathways (shown in Fig.1f).

File name: Supplementary Data 3
Description: Gene ontology enrichment analysis of DEGs identified between EPI and PE populations identified by scRNA-seq of E4.5 embryos in Mohammed et al., 2017 (sheet: GO_from_DEGs). Second sheet "GO_signaling_pathways" contains gene ontology enrichment analysis as in first sheet, narrowed down to signaling pathways (shown in Supplementary Fig. 1d).

File name: Supplementary Data 4
Description: Differential gene expression analysis between D8 *Tcf7l1* OE versus D0 cells. First sheet "Day8 UpRegulated" contains the genes upregulated at D8. Second sheet "Day8 Up Func Enrichments" contains gene ontology enrichment analysis from D8 upregulated genes (shown in Supplementary Fig. 4h). Third sheet "Day8 DownRegulated" contains the genes downregulated at D8. Fourth sheet "Day8 Down Func Enrichments" contains gene ontology enrichment analysis from D8 downregulated genes.

File name: Supplementary Data 5
Description: Differential gene expression analysis between D1, D2 and D4 *Tcf7l1* OE versus D0 cells. First "down_ESvsD1_pvaladj05" and second "up_ESvsD1_pvaladj05" sheets contain the list of downregulated and upregulated genes at D1, respectively. Third "down_ESvsD2_pvaladj05" and fourth "up_ESvsD2_pvaladj05" sheets contain the list of downregulated and upregulated genes at D2, respectively. Fifth "down_ESvsD4_pvaladj05" and sixth "up_ESvsD4_pvaladj05" sheets contain the list of downregulated and upregulated genes at D4, respectively.

File name: Supplementary Data 6
Description: ClueGO analysis on D1 downregulated genes upon *Tcf7l1* OE relating to Fig. 5a.

File name: Supplementary Data 7
Description: ClueGO analysis on D4 upregulated genes upon *Tcf7l1* OE relating to Fig. 5e.

File name: Supplementary Data 8
Description: List of genes bound by TCF7L1 obtained from the analysis of ChIP-seq data in De Jaime-Soguero et al., 2017 (first column). List of D1 downregulated genes upon *Tcf7l1* OE from this study (second column). The two lists of genes were overlapped and the intersection of bound and downregulated genes is presented on the third column. Relates to Fig. 5g.

File name: Supplementary Data 9
Description: Gene ontology enrichment analysis of the intersection of bound and downregulated genes (Supplementary Data 8). Relates to Fig. 5h.

File name: Supplementary Data 10
Description: Next generation sequencing data (MiSeq) of full frameshift *Tcf7l1* knock-out embryos. Exact type of mutation and sequence is shown. Relates to Fig. 6h-k.

File name: Supplementary Data 11
Description: Supplementary method information (primers, crRNA, MiSeq primers).
