# Supplementary material for: The Wnt/TCF7L1 transcriptional repressor axis drives primitive endoderm formation by antagonizing naive and formative pluripotency
Source: Nat Commun. 2023 Mar 3;14:1210. doi: 10.1038/s41467-023-36914-1 (PMC9984534; doi:10.1038/s41467-023-36914-1)
Supplement: Supplementary file 15 — Reporting Summary [file 41467_2023_36914_MOESM15_ESM.pdf]

Corresponding author(s): Frederic Lluis

Last updated by author(s): Feb 10, 2023

Reporting Summary

Nature Portfolio wishes to improve the reproducibility of the work that we publish. This form provides structure for consistency and transparency in reporting. For further information on Nature Portfolio policies, see our Editorial Policies and the Editorial Policy Checklist.

Statistics

For all statistical analyses, confirm that the following items are present in the figure legend, table legend, main text, or Methods section.

n/a	Confirmed	
☐	☒	The exact sample size (n) for each experimental group/condition, given as a discrete number and unit of measurement
☐	☒	A statement on whether measurements were taken from distinct samples or whether the same sample was measured repeatedly
☐	☒	The statistical test(s) used AND whether they are one- or two-sided<br>*Only common tests should be described solely by name; describe more complex techniques in the Methods section.*
☒	☐	A description of all covariates tested
☐	☒	A description of any assumptions or corrections, such as tests of normality and adjustment for multiple comparisons
☐	☒	A full description of the statistical parameters including central tendency (e.g. means) or other basic estimates (e.g. regression coefficient) AND variation (e.g. standard deviation) or associated estimates of uncertainty (e.g. confidence intervals)
☐	☒	For null hypothesis testing, the test statistic (e.g. F, t, r) with confidence intervals, effect sizes, degrees of freedom and P value noted<br>*Give P values as exact values whenever suitable.*
☒	☐	For Bayesian analysis, information on the choice of priors and Markov chain Monte Carlo settings
☒	☐	For hierarchical and complex designs, identification of the appropriate level for tests and full reporting of outcomes
☒	☐	Estimates of effect sizes (e.g. Cohen's d, Pearson's r), indicating how they were calculated

Our web collection on statistics for biologists contains articles on many of the points above.

Software and code

Policy information about availability of computer code

Data collection	Cultured cell images were taken by Leica DMi1 microscope. Realtime PCR results were collected by ViiA 7 Real-Time PCR system (AB applied biosystems). Confocal images were taken by Leica TCS SP8 confocal microscope (Leica). Immunofluorescence images were taken by Zeiss AxioImager Z1 Microscope (Zeiss).<br><br>RNA-seq, scRNA-seq and ChIP-seq data sets were processed and analyzed using the following tools:<br>PySCENIC v0.9.15<br>Seurat v3.0.0<br>Harmony v0.1.4<br>Palantir v1.0.0<br>Cutadapt<br>HISAT2 v2.1.0<br>FeatureCounts v2.0.1<br>DESeq2 v1.28.1<br>ClueGO Cytoscape plug-in<br>Metascape<br>GEM<br>g: Profiler<br>R v3.6.1<br>IGV Genome Browser

Data analysis	qPCR data were analyzed by QuantStudio™ Real-Time PCR Software v1.3. Statistic analysis were calculated by GraphPad Prism v6.0 unless stated otherwise. Immunofluorescence staining images and WB were adjusted by ImageJ.

For manuscripts utilizing custom algorithms or software that are central to the research but not yet described in published literature, software must be made available to editors and reviewers. We strongly encourage code deposition in a community repository (e.g. GitHub). See the Nature Portfolio guidelines for submitting code & software for further information.

Data

Policy information about availability of data

All manuscripts must include a data availability statement. This statement should provide the following information, where applicable:

- Accession codes, unique identifiers, or web links for publicly available datasets
- A description of any restrictions on data availability
- For clinical datasets or third party data, please ensure that the statement adheres to our policy

All data generated in this study are provided in the article file, Supplementary Information, Supplementary Data and Source Data files. The RNA-seq data generated in this study has been deposited in the Gene Expression Omnibus (GEO) database under the accession code: GSE171204. The publicly available TCF7L1 ChIP-seq data in mESCs used in this study are available in the ArrayExpress database under the accession code: E-MTAB-4358. The publicly available scRNA-seq data used for gene expression and SCENIC analysis are available in the GEO database under the accession codes: GSE145609, GSE100597 and GSE123046. Source data are provided with this paper.

Human research participants

Policy information about studies involving human research participants and Sex and Gender in Research.

Reporting on sex and gender	N/A
Population characteristics	N/A
Recruitment	N/A
Ethics oversight	N/A

Note that full information on the approval of the study protocol must also be provided in the manuscript.

Field-specific reporting

Please select the one below that is the best fit for your research. If you are not sure, read the appropriate sections before making your selection.

☒ Life sciences ☐ Behavioural & social sciences ☐ Ecological, evolutionary & environmental sciences

For a reference copy of the document with all sections, see nature.com/documents/nr-reporting-summary-flat.pdf

Life sciences study design

All studies must disclose on these points even when the disclosure is negative.

Sample size	Pilot experiments were performed to estimate the intrinsic variability of the experimental data and to determine the optimal sample size for a given experiment, suitable for detecting statistically significant differences. The exact sample size in each experiment is indicated in the corresponding figure legend.
Data exclusions	No data were excluded from the analysis.
Replication	Most experiments were performed using at least three biological replicates, except experiments shown in Fig. 3k and Fig. 5i which were performed once or twice. Ex vivo experiments were performed by conducting at least 2 in vitro culture sessions per experiment. All attempts at replication were successful.
Randomization	All embryos were randomly allocated to the experimental groups and used for the following experiments: antagonist and chemical inhibitor treatments, immunofluorescence analysis. For experiments other than those mentioned here, random allocation is not relevant. For example, for stem cell treatment or stem cell differentiation experiments the investigators need to know specific stem cell type, plating density, differentiation protocol and differentiation time and therefore random allocation was not performed for these experiments.
Blinding	To process experimental samples for downstream analysis, the Investigators needed to know the information regarding culture conditions, stem cell types and which method/protocol to use. The same investigator set up and analysed the experiments, no blinding was performed. Unbiased analysis of data was carried out wherever possible.

Reporting for specific materials, systems and methods

We require information from authors about some types of materials, experimental systems and methods used in many studies. Here, indicate whether each material, system or method listed is relevant to your study. If you are not sure if a list item applies to your research, read the appropriate section before selecting a response.

Materials & experimental systems

n/a	Involved in the study
☐	☒ Antibodies
☐	☒ Eukaryotic cell lines
☒	☐ Palaeontology and archaeology
☐	☒ Animals and other organisms
☒	☐ Clinical data
☒	☐ Dual use research of concern

Methods

n/a	Involved in the study
☒	☐ ChIP-seq
☐	☒ Flow cytometry
☒	☐ MRI-based neuroimaging

Antibodies

Antibodies used

Rat monoclonal anti-Nanog antibody (eBioscience™)
Goat polyclonal anti-Gata6 antibody (R&D systems)
Mouse monoclonal anti-Gata4 antibody (BD Biosciences)
Mouse monoclonal anti-Cdx2 antibody (Biogenex)
Rabbit monoclonal anti-(active)-β-catenin antibody (Cell Signaling Technology)
Goat polyclonal anti-Tcf3 (M-20) (Tcf7l1) antibody (Santa Cruz Biotechnology)
Goat polyclonal anti-Nestin (Santa Cruz Biotechnology)
Mouse monoclonal anti-β-catenin antibody (BD Biosciences)
Rabbit monoclonal anti-Tcf1 (C63D9) antibody (Cell Signaling Technology)
Rabbit monoclonal anti-Lef1 (C12A5) antibody (Cell Signaling Technology)
Mouse anti-β-Actin (C4) antibody (Santa Cruz)
PE anti-mouse CD140a (PDGFRA) (APA5) antibody (Thermo Fisher Scientific)
APC anti-mouse CD31 (PECAM-1) (390) antibody (Thermo Fisher Scientific)
PE Rat IgG2α, κ Isotype Clone R35-95 (RUO) antibody (BD Biosciences)
APC Rat IgG2α, κ Isotype (eBR2a) antibody (Thermo Fisher Scientific)
Goat anti-mouse IgG-HRP (Santa Cruz Biotechnology)
Goat anti-rabbit H&L (HRP) (Abcam)
Donkey anti-Goat IgG (H+L), Alexa Fluor 488 (Invitrogen)
Donkey anti-Rat IgG (H+L), Alexa Fluor 555 (Abcam)
Donkey anti-Rabbit IgG (H+L), Alexa Fluor 647 (Abcam)
Donkey anti-Mouse IgG (H+L), Alexa Fluor 647 (Invitrogen)
Donkey anti-Goat IgG (H+L), Alexa Fluor 647 (Invitrogen)

Validation

-anti-Nanog antibody (14-5761-80, eBioscience™): The antibody guarantee covers the use of the antibody for IF applications. The antibody has been referenced in 60 publications. https://www.thermofisher.com/antibody/product/Nanog-Antibody-clone-eBioMLC-51-Monoclonal/14-5761-80
-anti-Gata6 antibody (AF1700, R&D systems): The antibody guarantee covers the use of the antibody for IF applications. The antibody has been referenced in 36 publications. https://www.rndsystems.com/products/human-gata-6-antibody_af1700
-anti-Gata4 antibody (560327, BD Biosciences): The antibody guarantee covers the use of the antibody for IF applications. The antibody has been referenced in 2 publications. https://www.bdbiosciences.com/us/applications/research/stem-cell-research/endoderm-markers/mouse/purified-mouse-anti-gata4-l97-56/p/560327
-anti-Cdx2 antibody (AM392GP, Biogenex): The antibody guarantee covers the use of the antibody for IF applications. The antibody has been referenced in 7 publications. https://biogenex.com/product/anti-cdx-2/?v=d3dcf429c679
-anti-(active)-β-catenin antibody (8814, Cell Signaling Technology): The antibody guarantee covers the use of the antibody for IF and WB applications. The antibody has been referenced in 198 publications. https://www.cellsignal.com/products/primary-antibodies/non-phospho-active-b-catenin-ser33-37-thr41-d13a1-rabbit-mab/8814
-anti-Tcf3 (M-20) (Tcf7l1) antibody (sc-8635, Santa Cruz Biotechnology): The antibody guarantee covers the use of the antibody for IF applications. The antibody has been referenced in 9 publications. https://www.scbt.com/p/tcf-3-antibody-m-20
-anti-Nestin antibody (sc-21248, Santa Cruz Biotechnology): The antibody guarantee covers the use of the antibody for WB and IF applications. The antibody has been referenced in 13 publications. https://www.scbt.com/p/nestin-antibody-g-20
-anti-β-catenin antibody (610154, BD Biosciences): The antibody guarantee covers the use of the antibody for WB applications. The antibody has been referenced in 6 publications. https://www.bdbiosciences.com/us/applications/research/stem-cell-research/cancer-research/human/purified-mouse-anti--catenin-14beta-catenin/p/610154
-anti-Tcf1 (C63D9) antibody (2203, Cell Signaling Technology): The antibody guarantee covers the use of the antibody for WB applications. The antibody has been referenced in 73 publications. https://www.cellsignal.com/products/primary-antibodies/tcf1-tcf7-c63d9-rabbit-mab/2203
-anti-Lef1 (C12A5) antibody (2230, Cell Signaling Technology): The antibody guarantee covers the use of the antibody for WB applications. The antibody has been referenced in 108 publications. https://www.cellsignal.com/products/primary-antibodies/lef1-c12a5-rabbit-mab/2230
-anti-β-Actin antibody (sc-47778, Santa Cruz Biotechnology): The antibody guarantee covers the use of the antibody for WB applications. The antibody has been referenced in 10027 publications. https://www.scbt.com/p/beta-actin-antibody-c4

-anti-CD140a (PDGFRA) PE (APA5) antibody (12-1401-81, Thermo Fisher Scientific): The antibody guarantee covers the use of the antibody for flow cytometry applications. The antibody has been referenced in 47 publications. https://www.thermofisher.com/antibody/product/CD140a-PDGFRA-Antibody-clone-APA5-Monoclonal/12-1401-81
-anti-CD31 (PECAM-1) APC (390) antibody (17-0311-82, Thermo Fisher Scientific): The antibody guarantee covers the use of the antibody for flow cytometry applications. The antibody has been referenced in 80 publications. https://www.thermofisher.com/antibody/product/CD31-PECAM-1-Antibody-clone-390-Monoclonal/17-0311-82
-anti-Rat IgG2α PE, κ Isotype Clone R35-95 (RUO) antibody (553930, BD Biosciences): The antibody guarantee covers the use of the antibody for flow cytometry applications. https://www.bdbiosciences.com/eu/reagents/research/antibodies-buffers/immunology-reagents/anti-mouse-antibodies/cell-surface-antigens/pe-rat-igg2a-isotype-control-r35-95/p/553930
-anti-Rat IgG2α APC, κ Isotype (eBR2a) antibody (17-4321-81, Thermo Fisher Scientific): The antibody guarantee covers the use of the antibody for flow cytometry applications. The antibody has been referenced in 37 publications. https://www.thermofisher.com/antibody/product/Rat-IgG2a-kappa-clone-eBR2a-Isotype-Control/17-4321-81

Eukaryotic cell lines

Policy information about cell lines and Sex and Gender in Research

Cell line source(s)	WT ESC and Tcf7l1 KO ESC were previously generated (Merrill et al., 2004) and kindly provided by Brad Merrill. Tcf7 KO ESC were previously generated in our laboratory and reported in De Jaime-Soguero et al., 2017. Tcf7l1 OE ESC were described in Nishiyama et al., 2009 and purchased from NIA Mouse ES Cell Bank.
Authentication	Cell lines were authenticated by genomic PCR, RT-qPCRs and immunostaining.
Mycoplasma contamination	All cell lines were routinely checked for mycoplasma contamination and negative results were obtained.
Commonly misidentified lines (See ICLAC register)	None

Animals and other research organisms

Policy information about studies involving animals; ARRIVE guidelines recommended for reporting animal research, and Sex and Gender in Research

Laboratory animals	Mice strain: CD-1 and B6D2F1. Morulae collected from 3-4 months old female CD-1 mice were used for ex vivo treatment with Wnt antagonists or chemical molecules and downstream analysis (e.g. immunofluorescence, lumen volume measurement). Blastocysts collected from 3-4 months old female CD-1 were used for microinjection and as surrogate mothers. Zygotes collected from B6D2F1 mice were used for CRISPR/Cas9 components injection and blastocysts were analyzed further.
Wild animals	The study did not involve the use of wild animals.
Reporting on sex	Findings apply to both sexes. Mouse embryos collected for all experiments were not sequenced for sex determination and were assigned to experimental groups regardless sex.
Field-collected samples	The study did not involve the use of field-collected samples.
Ethics oversight	Ethics Committee at KU Leuven University (P170/2019) and Animal Ethics Committee of Ghent University Hospital (ECD 18-29).

Note that full information on the approval of the study protocol must also be provided in the manuscript.

Flow Cytometry

Plots

Confirm that:

☒ The axis labels state the marker and fluorochrome used (e.g. CD4-FITC).

☒ The axis scales are clearly visible. Include numbers along axes only for bottom left plot of group (a 'group' is an analysis of identical markers).

☒ All plots are contour plots with outliers or pseudocolor plots.

☒ A numerical value for number of cells or percentage (with statistics) is provided.

Methodology

Sample preparation	Cells were dissociated into single cells by Cell Dissociation Solution or Accuatase solution.
Instrument	Cells were analyzed using FACS BD Canto HTS or sorted using BD FACSAria™ III sorter
Software	Analyzed by Flowjo v10.7.1
Cell population abundance	A minimum of 10,000 cells from each sample were analyzed.

Gating strategy	Preliminary FSC/SCC gates were used to remove debris, doublets and other aggregated particles from the cell population. Gates based on negative (unstained or isotype control) and/or positive (singly stained for each marker) controls were used to identify the target populations.

☒ Tick this box to confirm that a figure exemplifying the gating strategy is provided in the Supplementary Information.
